# Eye Behavior Associated with Internally versus Externally Directed Cognition

**DOI:** 10.3389/fpsyg.2017.01092

**Published:** 2017-06-30

**Authors:** Mathias Benedek, Robert Stoiser, Sonja Walcher, Christof Körner

**Affiliations:** Institute of Psychology, University of GrazGraz, Austria

**Keywords:** internal attention, goal-directed cognition, eye-tracking

## Abstract

What do our eyes do when we are focused on internal representations such as during imagination or planning? Evidence from mind wandering research suggests that spontaneous shifts from externally directed cognition (EDC) to internally directed cognition (IDC) involves oculomotor changes indicative of visual disengagement. In the present study, we investigated potential differences in eye behavior between goal-directed forms of IDC and EDC. To this end, we manipulated the focus of attention (internal versus external) in two demanding cognitive tasks (anagram and sentence generation). IDC was associated with fewer and longer fixations and higher variability in pupil diameter and eye vergence compared to EDC, suggesting reduced visual scanning and higher spontaneous eye activity. IDC was further related to longer blinks, lower microsaccade frequency, and a lower angle of eye vergence. These latter changes appear conducive to attenuate visual input and thereby shield ongoing internal processes from external distraction. Together, these findings suggest that IDC is accompanied by characteristic eye behavior that reflects a decoupling of attention from external events and serves gating out visual input.

## Introduction

Even though our eyes are open for most of the time when awake, our attention is commonly directed to internal processes, thus disregarding the visual stimulation coming from our environment. This is not only true for spontaneous episodes of mind wandering, but for many goal-directed cognitive activities such as contemplation and imagination, as they are largely independent of sensory information (Raichle, 2010). Different lines of research suggest that internally directed cognition (IDC) implies a state of visual disengagement, where eye behavior is decoupled from irrelevant external events, which may contribute to shield an internal train of thoughts from external distractions (Smallwood et al., 2007). So far, most studies have only looked at spontaneous forms of IDC such as mind wandering or compared quite different internal and external cognition tasks (e.g., Singer et al., 1971; Reichle et al., 2010). This limits comparability of cognitive processes and complicates the interpretation of differences in eye parameters. Therefore, the present study manipulated the direction of attention (external vs. internal) within goal-directed thinking tasks in order to examine the specific oculomotor behavior associated with goal-directed internal cognition.

Attention is a core cognitive function responsible for the selection of relevant information and maintenance of focus. A general distinction can be made between cognition characterized by externally directed versus internally directed attention, or briefly between externally directed cognition (EDC) versus IDC (Dixon et al., 2014). EDC involves the processing of attended external stimuli such as in reading or searching one’s visual environment. In contrast, IDC involves constructive processes that build on memory rather than sensory input to generate novel mental representations (Andrews-Hanna et al., 2014). Since these processes are largely independent from external stimulation they have also been labeled as stimulus-independent thought or self-generated thought (Andrews-Hanna et al., 2014; Christoff et al., 2016). Examples of IDC include planning, mental simulation, imagination, and more specifically thinking about the past or the future, thinking about the self or others, and creative idea generation.

Externally directed cognition and IDC are typically considered competing states due to limited conscious information processing capacity (Chun et al., 2011). When we try to focus on either external or internal stimuli, information from other sources may interfere. Yet, complex cognitive activities can also represent mixtures of EDC and IDC, meaning that they co-occur in an alternating fashion and, at lower levels of intentionality, may even cooperate (Dixon et al., 2014). Importantly, IDC (and EDC) can involve deliberate or spontaneous processing (Dixon et al., 2014). Spontaneous IDC occurs when we are at rest, or when attention is unintentionally drawn away from a task as during episodes of mind wandering (Kane et al., 2007; Smallwood and Schooler, 2015). In contrast, deliberate IDC is a goal-directed activity that essentially relies on internally directed attention (e.g., generating ideas, or performing mental arithmetic). The successful performance of demanding IDC thus requires that we stay focused on ongoing internal processes and not get distracted by external stimulation.

Available evidence suggests that spontaneous IDC is associated with specific changes in eye behavior that are indicative of visual disengagement. Early studies found that visual imagery and daydreaming are associated with higher frequencies of saccades and eye blinks (Antrobus et al., 1964; cf. Singer et al., 1971). However, these findings have received little attention in the vision literature, which seemed to be more concerned with the processing of external information (Ehrlichmann and Micic, 2012). More recently, examinations of mind wandering episodes during reading found that IDC is associated with fixations that are longer and less affected by the linguistic variables of the text (Reichle et al., 2010; Uzzaman and Joordens, 2011). Another study found that mind wandering is accompanied by increased variability of pupil diameter (PD), suggesting a decoupling of attention from the external task (Smallwood et al., 2011). Finally, mind wandering has been associated with smaller baseline PD (Grandchamp et al., 2014; Unsworth and Robison, 2016). Together these findings provide evidence that oculometric parameters are sensitive to spontaneous shifts of the attentional focus away from an external task. These oculometric changes may reflect a reduced responsiveness to external stimulation (Smallwood et al., 2011) or even a coupling to relevant internal events (Ferreira et al., 2008).

Further support for the important role of eye behavior in IDC comes from the study of gaze aversion. Gaze aversion refers to the aversion of one’s eyes (or even brief eye closure) during demanding processes requiring internal attention. There is strong evidence that gaze aversion serves the function of reducing cognitive load during demanding cognitive activities (e.g., mental arithmetic) by avoiding the processing of potentially distracting external stimuli in order to shield internal processes (Doherty-Sheddon and Phelps, 2005; Markson and Paterson, 2009). Gaze aversion was shown to enhance visual imagination (Vredeveldt et al., 2011; Buchanan et al., 2014) and retrieval (Glenberg et al., 1998), especially during face-to-face interactions. Similarly, a recent eye tracking study found that insight solutions are preceded by longer blink durations and gazing away from the stimulus, which was interpreted as a shutting out or interruption of visual input in moments of insight (Salvi et al., 2015; Salvi and Bowden, 2016). These findings indicate that eye behavior may also actively support IDC by means of reducing visual information processing to avoid interference by sensory stimulation, and this shielding function may be particularly relevant for sustained forms of goal-directed IDC.

Averting one’s gaze from salient external stimuli or eye closure are straightforward ways to reduce cognitive load from visual input. Another potential mechanism would include the release of visual focus in terms of disaccommodation. The ocular mechanism related to visual accommodation to a certain near distance is well understood: It is achieved by a response complex called the *near triad*, which includes the bending of the lens, concurrent eye convergence (i.e., an adaptation of the angle of eyes), and pupillary constriction (Myers and Stark, 1990). Visual disaccommodation from a near focus hence can be assumed to involve an inverse pattern, consisting of the divergence of eyes and pupillary dilation. Eye vergence (as well as pupillary diameter) can be readily assessed by means of eye tracking (Solé Puig et al., 2013) and therefore could represent another accessible oculometric indicator of visual disengagement. Similarly, attenuation of visual perception could also be achieved by means of reduced microsaccade activity. When fixating static stimuli, neuronal adaptation leads to perceptual fading within seconds, unless it is counteracted by brief fixational eye movements (i.e., microsaccades; Martinez-Conde et al., 2006, 2013; McCamy et al., 2012). Eye vergence and microsaccade activity hence qualify as indicators of visual disengagement, and therefore are considered as further relevant eye parameters in this research.

The findings reviewed above indicate that IDC is characterized by visual disengagement and hence by changes of eye parameters. So far, most of the available research has focused on spontaneous types of IDC, where attention is inadvertently drawn away from an external task. However, little is known about goal-directed forms of IDC that require sustained internally focused attention over longer time periods and thus should be particularly prone to interference from the external world. Therefore, in the present study we investigated the oculometric profile associated with goal-directed IDC compared to EDC. In order to avoid effects associated with task differences between EDC and IDC tasks, we manipulated the direction of attention within the same task. According to the literature on spontaneous perceptual decoupling, internal cognition should be associated with increased variability of PD (Smallwood et al., 2011), longer fixation durations (Reichle et al., 2010), and smaller PD (Grandchamp et al., 2014; Unsworth and Robison, 2016). Moreover, active forms of visual disengagement supporting the attenuation of visual information processing may include more or longer blinks (Salvi et al., 2015), potentially reduced microsaccade activity (Martinez-Conde et al., 2013), and visual disaccommodation in terms of reduced angle of eye vergence (Myers and Stark, 1990; Solé Puig et al., 2013).

## Materials and Methods

### Participants

The final sample consisted of 46 young adults, aged between 18 and 33 years (*M* = 23.3, *SD* = 4.0; 65% female, 33% male, 2% other gender identity). Four additional participants were excluded from all analyses due to technical reasons such as unreliable calibration. All participants had normal or corrected-to-normal (soft contact lenses) vision, reported no strabismus or other medical condition affecting vision. They participated for partial course credit and the possibility to take part in a raffle. All participants gave written informed consent. The study was approved by the local ethics committee of the Karl-Franzens-University of Graz, Austria.

### Experimental Tasks and Procedure

Participants worked on anagram (AN) and sentence generation (SG) tasks, which were both performed in an *internal* attention and an *external* attention condition. These tasks were selected because both tasks generally rely on externally directed attention, but can also be performed reasonably well in the mind’s eye (Benedek et al., 2011). This allows for a subtle experimental manipulation of the direction of attention within the same tasks. Using two tasks per condition further enables to test the consistency of attention effects across different task types (i.e., convergent and divergent thinking; Benedek et al., 2011). This experimental protocol has been used in previous research examining the neural correlates of IDC (Benedek et al., 2011, 2014, 2016), but so far it has not been properly examined with eye tracking.

Stimuli in both tasks were meaningful, German four-letter words (e.g., “POST”). In the AN task, participants were required to rearrange all four letters of the stimulus to find a new, meaningful word (e.g., “STOP”). In the SG task, participants were required to generate an original, meaningful sentence by using the four stimulus letters as initial letters (e.g., “Oldies sometimes provoke tears”). In both tasks every single letter of the stimulus word had to be used exactly once, regardless of the sequence. In the *external* condition, the stimulus word remained on screen throughout the task, whereas in the *internal* condition the stimulus was masked after a brief initial encoding period. The internal condition hence enforced internally directed attention as the task was performed “in the mind’s eye.”

Specifically, in every trial, the stimulus word was presented in black capital letters in the center of a gray screen [RGB = 204,204,204]. In the external condition, the stimulus was presented for 20 s, whereas in the internal condition the stimulus was shown for only 0.5 s, and then became masked by “XXXX” for the remaining 19.5 s (preventing access to the stimulus, while ensuring similar visual stimulation as in the external condition). During this task period, participants had to find a solution and keep their gaze on the center of the screen. In the case that participants came up with a response before the 20 s elapsed, they were instructed to keep thinking about further potential anagram solutions, or about more original sentences to ensure constant task-related activity within the entire task period. After this task period, the stimulus word appeared in green letters for 6 s, prompting the participants to vocalize their solution. The responses were recorded by the experimenter to verify that participants paid close attention to the tasks.

Participants received thorough task instructions explaining the two different tasks followed by eight practice trials. The experiment included 36 trials (18 AN trials, and 18 SG trials). Trials were grouped into 6 blocks à 6 trials of the same task to reduce task switching efforts. The task blocks were ordered in an ABBAAB or BAABBA fashion. Each block started with a task cue (5 s) indicating the task to be performed in this block (“Anagram” or “Sentence generation”). The cue was followed by 6 trials, half from the external, and half from the internal condition. The sequence of external and internal trials was randomized. Trials were separated by 2 s of a blank screen followed by the brief presentation of a fixation disk for the duration of the drift correction of the eye tracker. The total experiment took about 20 min.

### Apparatus

Participants were placed in a sound attenuated room with the lights turned on and sat at a distance of 50 cm from the screen. Their heads were stabilized using chin rest and forehead rest of the EyeLink Tower Mount (SR Research, Ottawa, ON, Canada). Stimuli were presented on a 19″ LG flatron L1920P monitor run at 60 Hz and at 1280 × 1024 pixels resolution, subtending 29.4 pixels per degree visual angle (v.a.). Binocular eye data were recorded using an EyeLink 1000 Plus Tower Mount eye tracker (SR Research, Ottawa, ON, Canada) with a temporal resolution of 500 Hz. For stimulus presentation and response recording, the EyeLink Experiment Builder software (SR Research, Ottawa, ON, Canada) was used. For calibration, validation, drift correction, and computation of the eye movement parameters (blinks, fixations, and saccades), we used the manufacturer’s software (SR Research, Ottawa, ON, Canada). For saccade detection, the velocity threshold was set to 35°/s and the acceleration threshold to 9,500°/s^2^. There was a 9-point calibration procedure at the beginning of the experiment and a drift correction before each trial. Spatial resolution was typically better than 0.30° v.a.. Participants’ answers were recorded with a microphone to monitor and assess task performance.

### Data Analysis

The analysis of eye parameters focused on the final 18.5 s of each trial, thus excluding data of the initial 1.5 s that might be affected by the conditional stimulus masking after 0.5 s. Blinks were automatically detected by the eye tracking software (SR Research, Ottawa, ON, Canada) and removed from gaze position and PD data. Only data for which the eye tracker had recorded both eyes were analyzed. Fixation durations, fixation counts, blinks and saccades and saccade amplitude per trial were calculated with Data Viewer (SR Research, Ottawa, ON, Canada). Further data analyses were performed using R^1^. For calculation of PD and eye vergence, eye tracking data were down-sampled from 500 to 50 Hz by averaging across 10 data points (20 ms). Pupil diameter was defined as the average across both eyes and was z-transformed. Calculation of angle of eye vergence (AoEV) was similar to methods applied in previous research (Solé Puig et al., 2013). Using participants’ individual inter-pupil distance (measured with a transparent ruler), gaze positions of both eyes and the distance of the screen to the observer (50 cm), gaze vectors for each eye were calculated. Gaze position coordinates were transformed from pixels to mm (3.4 pixels per mm). Gaze positions with fixation disparities outside the margins of participants’ pupil distance plus 10 mm in both sides (negative and positive fixation disparity) were removed as artifacts, as fixation disparities of this size do not occur during normal gaze behavior of healthy adults (0.2% of data). The intersection point (or closest approximation if vectors did not intersect) of the right and left gaze vectors was calculated with the function qr.solve of the {base} R-package^1^. The distance of the intersection point from midpoint between eyes was used as length of gaze vector. AoEV in degrees was then calculated with the following formula (the mean inter-pupil distance was set to 60 mm):

$$\begin{array}{c} AoEV=2*\mathrm{atan}(\frac{pupildistance/2}{lengthofgazevector})*\frac{180}{\pi } \end{array}$$

From the continuous pupil and AoEV data we finally computed the arithmetic mean and variance per trial to obtain separate scores for the central tendency and variability of these measures (cf. Smallwood et al., 2011). For computation of microsaccade activity, original 500 Hz gaze position data were used. Blinks were removed as well as additional 200 ms periods before and after each blink to eliminate parts where the pupil was partially occluded (McCamy et al., 2012). Microsaccades (count and amplitude) were determined using the Microsaccade Toolbox for R (Engbert et al., 2015) with microsaccades defined as saccades with an amplitude smaller than 1.0°, a minimum duration of 6 ms, and λ = 4 (McCamy et al., 2012). Only binocular microsaccades (i.e., with a minimum overlap of one data sample) were considered. Microsaccade measures were averaged across both eyes. Finally, for all oculometric parameters, we computed mean scores for each of the four experimental conditions. In order to ensure robust scores, we discarded trials with less than 50% valid data (0.2% of trials) as well as outliers (i.e., more than three standard deviations from the individual mean; 1.1% of trials) before averaging across relevant trials. The main analyses included both correctly and incorrectly solved trials as we assume that the latter involved similar cognitive processes but simply were not terminated in time; concurrent analyses limited to correct trials yielded essentially the same results.

## Results

### Task Performance

The average solution rate across experimental conditions and tasks was 77.84%, suggesting that the tasks were cognitively demanding yet solvable within the given task duration. Differences in task performance between EDC and IDC and the two tasks (AN versus SG) were tested with a two-way repeated measures ANOVA. The AN task turned out to be easier than the SG task (AN: *M* = 83.5%, *SE* = 1.4; SG: *M* = 72.2%, *SE* = 3.0; *F*[1,45] = 14.27, *p* < 0.01, $\eta_{p}^{2}$ = 0.24). As expected, the solution rate was higher in the external attention condition (*M* = 81.8%; *SE* = 2.0) compared to the internal attention condition (*M* = 73.9%; *SE* = 2.2; *F*[1,45] = 12.87, *p* < 0.01; $\eta_{p}^{2}$ = 0.22), as the latter condition required that tasks are performed in the mind’s eye. The attention manipulation tended to have a lower effect on AN task performance (EDC: *M* = 85.51%; *SE* = 1.8 vs. IDC: *M* = 81.40%; *SE* = 2.2) than on SG task performance (EDC: *M* = 78.02%; *SE* = 3.2 vs. IDC: *M* = 66.43; *SE* = 3.5; attention condition × task - interaction: *F*[1,45] = 4.05, *p* = 0.05, $\eta_{p}^{2}$ = 0.08).

### Oculometric Results

**FIGURE 1:**
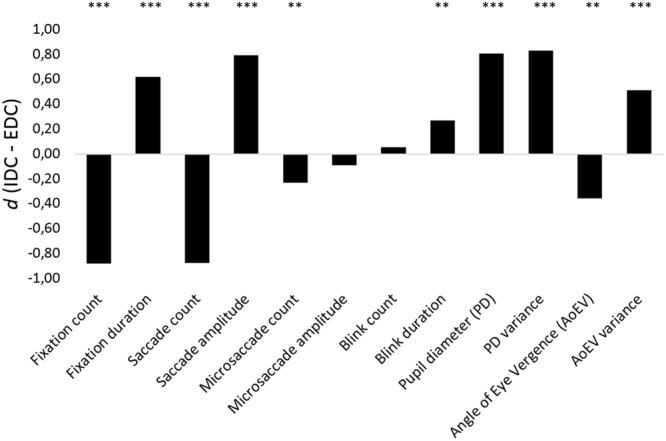
Effect sizes of oculometric differences between internally directed cognition (IDC) and externally directed cognition (EDC); ^∗∗^ *p* < 0.01, ^∗∗∗^ *p* < 0.001.

**Table 1** presents the results for the oculometric parameters separately for EDC and IDC and for both tasks (AN and SG). Differences between attention conditions and tasks were tested with two-way ANOVAs for each oculometric parameter. Looking first at attention effects, IDC involved a lower fixation count (*F*[1,45] = 146.62, *p* < 0.001, $\eta_{p}^{2}$ = 0.77), higher fixation duration (*F*[1,45] = 44.87, *p* < 0.001, $\eta_{p}^{2}$ = 0.50), lower saccade count (*F*[1,45] = 145.15, *p* < 0.001, $\eta_{p}^{2}$ = 0.76), higher saccade amplitude (*F*[1,45] = 28.57, *p* < 0.001, $\eta_{p}^{2}$ = 0.39), and lower microsaccade count (*F*[1,45] = 7.78, *p* = 0.008, $\eta_{p}^{2}$ = 0.15) than EDC, but attention conditions did not differ in the average microsaccade amplitude (*F*[1,43] = 0.39, *p* = 0.54), or blink count (*F*[1,45] = 1.13, *p* = 0.29). IDC further produced a higher blink duration (*F*[1,44] = 7.54, *p* = 0.009, $\eta_{p}^{2}$ = 0.15), higher PD (*F*[1,45] = 78.52, *p* < 0.001, $\eta_{p}^{2}$ = 0.64) as well as higher PD variance (*F*[1,45] = 88.12, *p* < 0.001, $\eta_{p}^{2}$ = 0.66), smaller AoEV (i.e., accommodation at higher distance; *F*[1,45] = 9.37, *p* = 0.004, $\eta_{p}^{2}$ = 0.17), and higher AoEV variance (*F*[1,45] = 17.03, *p* < 0.001, $\eta_{p}^{2}$ = 0.28). Effect sizes (*d*, corrected for paired-sample tests; Dunlap et al., 1996) of all oculometric differences between IDC and EDC are displayed in **Figure 1**.

**Table 1:** Mean (and SE) for each oculometric parameter during externally directed cognition (EDC) versus internally directed cognition (IDC) for both tasks.

	EDC			IDC		
	AN	SG	Total	AN	SG	Total
Fixation count [1/min]	122.19 (4.65)	121.39 (4.70)	121.79 (4.52)	88.41 (4.64)	98.78 (5.29)	93.59 (4.84)
Fixation duration [ms]	497.14 (25.92)	482.97 (24.35)	490.06 (24.36)	761.63 (60.64)	649.18 (43.06)	705.40 (48.28)
Saccade count [1/min]	119.60 (4.66)	118.99 (4.73)	119.29 (4.54)	85.90 (4.67)	96.45 (5.30)	91.17 (4.86)
Saccade amplitude [degree]	0.88 (0.04)	0.91 (0.05)	0.90 (0.04)	1.41 (0.11)	1.68 (0.18)	1.54 (0.14)
Microsaccade count [1/min]	26.45 (5.92)	22.86 (5.43)	24.65 (5.56)	15.69 (4.20)	16.98 (5.00)	16.33 (4.58)
Microsaccade ampl. [degree]	0.79 (0.12)	0.74 (0.04)	0.82 (0.12)	0.72 (0.06)	0.78 (0.06)	0.76 (0.06)
Blink count [1/min]	22.75 (2.17)	29.39 (2.89)	26.07 (2.49)	25.44 (2.31)	28.75 (2.77)	27.01 (2.49)
Blink duration [ms]	112.34 (5.11)	111.60 (5.06)	111.91 (4.90)	131.20 (9.49)	123.85 (7.85)	126.84 (8.55)
Scaled PD	–0.53 (0.03)	0.19 (0.04)	–0.17 (0.02)	–0.18 (0.04)	0.48 (0.04)	0.15 (0.02)
PD variance	0.33 (0.02)	0.35 (0.02)	0.34 (0.02)	0.50 (0.03)	0.42 (0.02)	0.46 (0.02)
AoEV [degree]	6.83 (0.02)	6.82 (0.03)	6.83 (0.02)	6.77 (0.02)	6.76 (0.03)	6.77 (0.03)
AoEV variance [degree^2^]	0.07 (0.01)	0.07 (0.01)	0.07 (0.01)	0.09 (0.01)	0.10 (0.01)	0.10 (0.01)

*AN, anagram task; SG, sentence generation task; PD, pupil diameter; AoEV, angle of eye vergence.*

The observed attention effects were highly consistent across AN and SG tasks. No attention condition by task interaction effects were observed for microsaccade count (*F*[1,45] = 3.97, *p* = 0.05), PD (*F*[1,45] = 1.00, *p* = 0.32), AoEV (*F*[1,45] = 0.00, *p* = 0.96), and AoEV variance (*F*[1,45] = 0.60, *p* = 0.44). Significant interaction effects in other parameters (fixations count: *F*[1,45] = 25.07, *p* < 0.001, $\eta_{p}^{2}$ = 0.36; fixation duration: *F*[1,45] = 6.99, *p* = 0.01, $\eta_{p}^{2}$ = 0.13; saccade count: *F*[1,45] = 24.80, *p* < 0.001, $\eta_{p}^{2}$ = 0.36; saccade amplitude: *F*[1,45] = 5.77, *p* = 0.02, $\eta_{p}^{2}$ = 0.11; blink duration: *F*[1,45] = 8.45, *p* = 0.006, $\eta_{p}^{2}$ = 0.16; PD variance: *F*[1,45] = 20.72, *p* = 0.004, $\eta_{p}^{2}$ = 0.31) mostly reflected that attention effects were more pronounced in the AN than in the SG task, but still significant for both tasks separately (all *p*s < 0.05). Additionally, we observed significant interaction effects (but no main effects of attention condition) for blink count (*F*[1,45] = 15.03, *p* < 0.001, $\eta_{p}^{2}$ = 0.25), indicating that IDC was related to higher blink counts only in the AN task (*t*[45] = 3.36, *p* = 0.002) but not the SG task (*t*[45] = -0.71, *p* = 0.48).

Finally, ANOVAs also yielded significant task effects for various oculometric parameters. Most of them were driven by the interaction effects and cannot be interpreted globally, because these parameters only differed significantly during IDC but not during EDC. Independent task effects were only observed for PD (*F*[1,45] = 161.12, *p* < 0.001, $\eta_{p}^{2}$ = 0.78) and blink count (*F*[1,45] = 22.71, *p* < 0.001, $\eta_{p}^{2}$ = 0.34), which were significantly higher in the SG task than in the AN task in both attention conditions.

## Discussion

This study compared the oculomotor behavior associated with goal-directed IDC and EDC. Performance of the very same task either with access to a relevant visual stimulus or in the mind’s eye differed substantially in most of the observed oculometric parameters. Not surprisingly, during IDC people showed a reduced frequency of fixations and saccades, while the fixation duration and the saccade amplitude increased. These differences indicate that EDC involves a much more intense scanning of relevant visual information (i.e., the letters in the presented word stimulus) as compared to IDC, where no relevant visual information was available. This is consistent with research on mindless reading, reporting fewer fixations and higher fixation durations during mind wandering episodes compared to normal reading (Reichle et al., 2010; Uzzaman and Joordens, 2011). As another finding, eye behavior became more variable during IDC in terms of higher PD variance and AoEV variance. This result is in line with a study by Smallwood et al. (2011), who observed that more variable PD predicted encoding failures and slow responses in a choice-reaction time task, and thus could be indicative of mind wandering episodes during task performance. These findings suggest that eye movements become less guided and more spontaneous during IDC.

Internally directed cognition was also associated with longer blinks, reduced microsaccade counts, and a reduced AoEV compared to EDC. These oculometric changes are conducive to the attenuation of visual input in different ways. First, an increased average blink duration obviously reduces the total time of visual perception. Salvi et al. (2015) also observed longer average blink durations in a 2-s period prior to the solution when word problems were solved with insight rather than analytically. They concluded that insight solutions might be supported by a transient shutting out of irrelevant visual input. Second, microsaccade activity during fixation counteracts perceptual fading (Martinez-Conde et al., 2006, 2013; McCamy et al., 2012). The reduced microsaccade frequency during IDC thus may implicate higher perceptual fading. Finally, the decrease in the AoEV indicates that the visual focus moved to a farer distance, a state which has been captured by the colloquial term of “staring into space.” The disaccommodation from available visual stimuli (i.e., the screen in front of participants) during IDC hence undermines perception. Together, longer blinks, fewer microsaccades and divergence of eyes are indicators of reduced visual processing. It is possible that these changes are partly due to differences in visual stimulation (i.e., meaningless versus meaningful four-letter words) and a reduced necessity to process this visual information, but they may also represent more active mechanisms to shield internal representations from external distraction. Gaze aversion and eye closure are other well-known strategies to reduce cognitive interference from visual stimulation during demanding IDC and effectively increase task performance (Doherty-Sheddon and Phelps, 2005; Markson and Paterson, 2009). Future research should investigate whether blink duration, microsaccade activity and eye divergence can in fact be functionally linked to an effective gating of distractors as well as to higher performance.

The observed effects were highly consistent across two different tasks. Moreover, effects are also largely consistent with findings from another recent study, which compared eye behavior between an external reading task and an internal idea generation task while presenting identical visual displays (Walcher et al., in press). In that study IDC was also associated with higher blink duration (and higher blink frequency), lower microsaccade counts and higher pupil dilation compared to EDC. Both studies thus provide evidence that IDC is associated with gating-related eye behavior (i.e., longer blinks and fewer microsaccades), and these effects seem to hold for manipulations of IDC versus EDC within the same cognitive task (as in the present study) as well as for different tasks but identical visual displays (as in Walcher et al., in press). As a notable difference between studies, however, idea generation involved more fixations than letter reading, whereas in the present study IDC was associated with less fixations than EDC. These findings corroborate the view that fixation counts strongly depend on the type of external task and thus may not be a good indicator of the direction of attention: fixations can be reduced when attention is bound on a single spot as in letter reading, or increased when the task requires to scan different characters.

These oculometric effects lend broad support to the perceptual decoupling hypothesis, which posits that IDC involves a specific state of mind, where attention is decoupled from sensory information (Frith and Frith, 2006; Raichle, 2010; Smallwood, 2013). Part of the oculometric effects (e.g., lower fixation counts and higher oculomotor variability) indicate that eye behavior is no longer tied to predictable external cues or tasks during IDC, but instead varies spontaneously or even becomes coupled to internal events (Ferreira et al., 2008). Other oculometric changes such as higher blink durations, reduced microsaccade activity, and lower AoEV may be more directly geared toward the attenuation of the visual input. Reducing the stream of visual information represents a straightforward oculomotor mechanism to shield the ongoing internal train of thought from external distractions.

While findings were mostly consistent across tasks, two eye parameters appeared sensitive to task demands: PD and blink rates were increased during SG compared to the AN task. Increased PD and blink rate are common indicators of cognitive load and task difficulty (Kahneman and Beatty, 1966; Porter et al., 2007; Siegle et al., 2008). These task effects thus may be attributed to the slightly higher task difficulty in the SG task, as evidenced by lower solution rates. This might partly explain why PD was generally higher during IDC, because internal processing was slightly more difficult than external processing. It might also explain the discrepancy with previous research associating mind wandering with lower PD (Grandchamp et al., 2014; Unsworth and Robison, 2016), because mind wandering or lapses of attention seem related to lower attentional control whereas goal-directed IDC was related to increased attentional demands. We do not believe, however, that the observed attention effects for other oculometric parameters are generally due to differences in task difficulty, as they are not known to be primarily sensitive to task difficulty (but see, Gao et al., 2015).

How do the present oculometric findings relate to neuroscientific evidence on IDC? EEG research shows that IDC is consistently associated with increased EEG alpha power especially at posterior brain regions (Ray and Cole, 1985; Fink and Benedek, 2013, 2014). This alpha synchronization effect applies to different forms of IDC including creative idea generation (Fink et al., 2009; Jauk et al., 2012), insight problem solving (Kounios and Beeman, 2014), imagery (Cooper et al., 2003; Bartsch et al., 2015), and memory maintenance (Klimesch, 2012), and has also been observed in within-task manipulations of internal versus external attention (Benedek et al., 2011, 2014). Moreover, a recent fMRI study employing the same experimental design as the present study found that IDC versus EDC is associated with substantially reduced brain activation in extended regions of the occipital cortex (Benedek et al., 2016). EEG research on mind wandering provides further evidence for reduced cortical activity in regions associated with sensory processing (Smallwood et al., 2008; Baird et al., 2014). Together, these neurophysiological findings suggest that IDC involves a reduced processing of visual information. Considering the present oculometric findings, the reduced brain activation in visual networks hence could be due to the effective perceptual decoupling and visual gating mechanisms at the oculomotor level. For example, EEG alpha synchronization is typically strongest over occipital regions when eyes are closed (Legewie et al., 1969). As an alternative explanation, however, neurophysiological effects could represent an independent top-down mechanism to suppress visual information processing at a neural level during demanding internal cognition. EEG alpha activity was shown to increase with memory load (Jensen et al., 2002), and especially contralateral to unattended visual space (Rihs et al., 2007), which advocates a more active role of alpha activity in terms of top-down inhibition of task-irrelevant brain regions (Jensen et al., 2012; Klimesch, 2012). Further support for the top-down account comes from brain connectivity analyses showing that frontal and parietal regions exhibit increased functional connectivity with occipital areas during IDC (Sauseng et al., 2005; Benedek et al., 2016). For example, the right supramarginal gyrus showed increased functional connectivity with extended occipital areas that actually reduced brain activation during IDC, which potentially represents top-down suppression of visual information processing (Benedek et al., 2016). Reduced brain activation in the visual cortex during IDC hence could be viewed as an effect of visual disengagement at oculomotor level, or as a complemental top-down mechanism at neural level (cf. Benedek, in press). This question should be addressed in future research by combining oculometric and neurophysiological assessments.

### Conclusion and Future Directions

Many cognitive activities such as planning and imagination require sustained internally directed attention. This study revealed that demanding IDC is accompanied by a characteristic oculomotor response reflecting different forms of visual disengagement. Reduced fixations and higher variability of oculomotor parameters suggest that eye behavior decouples from external stimuli during goal-directed IDC. Increased blink durations, reduced microsaccade activity and divergence of eyes seemed to target an active attenuation of visual information uptake. Such an oculomotor response may support demanding IDC by shielding ongoing internal information processing from external distraction. If this oculomotor response associated with internal cognition proves reliable in future research, it could serve as an objective indicator of the actual focus of visual attention: is a person looking at an external object or just looking in its direction while thinking about something else? Notably, the absence of external visual attention may not always imply an internal focus of attention, as attention might also be focused on other sensory modalities competing for the same attentional resources (e.g., trying to focus on some auditory input may also attenuate visual attention). Yet, it should be possible to infer whether attention is currently focused on the external visual environment or not.

Such an index of visual attention would be extremely helpful for future research on spontaneous and goal-directed internal versus external cognition, as well as for the study of transient shifts of attention during more complex cognitive activities that rely on both internal and external sources of information (cf. Dixon et al., 2014). For example, when following a lecture, looking at slides and listening to speakers generally involves externally directed attention, but actively processing this information requires temporary shifts of attention to internal processes in order to reconcile the new information with one’s knowledge base and generating own thoughts related to it. Effective learning likely involves a well-balanced assignment of attentional capacities to manage the steady stream of external and internal information without losing thread and eventually zoning out. Further possible applications of a time-sensitive oculometric index of visual attention include the improvement of driver monitoring systems, or the development of advertising applications that recognize whether they have effectively attracted attention. Eye-tracking thus is not only helpful to determine the direction of gaze, but to decide whether available visual information is consciously attended to or not.

## Author Contributions

MB, RS, SW, and CK planned the research. RS and SW carried out and analyzed the study. MB, RS, SW, and CK wrote the manuscript.

## Conflict of Interest Statement

The authors declare that the research was conducted in the absence of any commercial or financial relationships that could be construed as a potential conflict of interest.

## Footnotes

1 www.r-project.org
